# Supplementary figures and images for: Genome-wide identification of major genes and genomic prediction using high-density and text-mined gene-based SNP panels in Hanwoo (Korean cattle)
Source: PLoS One. 2020 Dec 2;15(12):e0241848. doi: 10.1371/journal.pone.0241848 (PMC7710051; doi:10.1371/journal.pone.0241848)

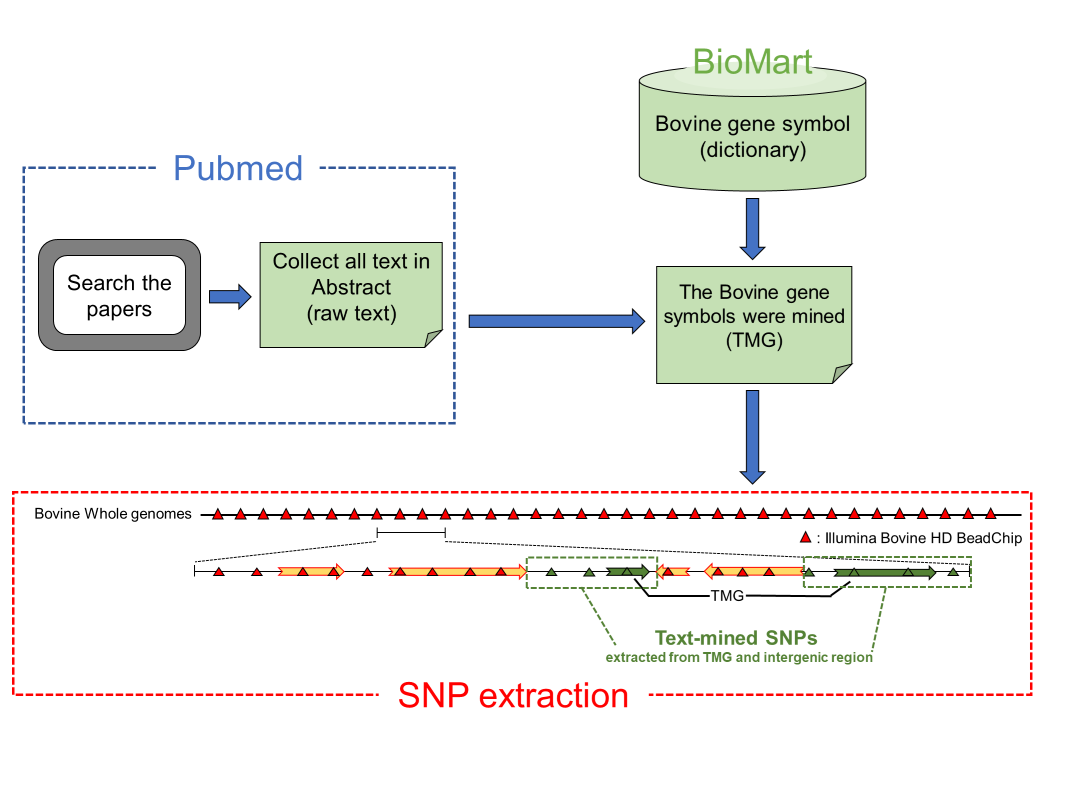

Supplement: S1 Fig — (TIF) [file pone.0241848.s001.tif]
