# Supplementary material for: Genome-wide identification of major genes and genomic prediction using high-density and text-mined gene-based SNP panels in Hanwoo (Korean cattle)
Source: PLoS One. 2020 Dec 2;15(12):e0241848. doi: 10.1371/journal.pone.0241848 (PMC7710051; doi:10.1371/journal.pone.0241848)
Supplement: Attachment — Submitted filename: Response_to_reviewer-Final.docx [file pone.0241848.s004.docx]

Reviewer #1: The authors have followed my recommendations to improve the manuscript. I suggest to add the information of tables and figures in point by point response to the manuscript and discuss the newly added results as well.

Thank you for this suggestion. As suggested by the reviewer, we added the evenly_SNPs + except results at table 6.

**Table 6. Accuracy of evenly-mined GBLUP and text-mined GBLUP**

| **Traits** | **exp_777k + tm_SNPs** | **exp_777k + em_SNPs^1^** |
| --- | --- | --- |
| **CWT** | 0.451 ± 0.01 | 0.471 ± 0.01 |
| **BF** | 0.421 ± 0.01 | 0.419 ± 0.01 |
| **EMA** | 0.437 ± 0.004 | 0.438 ± 0.01 |
| **S_SF** | 0.129 ± 0.03 | 0.099 ± 0.02 |
| **D_SF** | 0.189 ± 0.03 | 0.095 ± 0.02 |
| **S_IMF** | 0.168 ± 0.02 | 0.147 ± 0.02 |
| **D_IMF** | 0.225 ± 0.02 | 0.202 ± 0.03 |

**exp_777k + em_SNPs^1^**: multi-GRM GBLUP with evenly-mined SNPs and except SNPs.

These results have shown, in CWT, em_SNPs can make more accurate prediction than tm_SNPs. As with the results using whole SNPs, The polygenic characteristic of traits seem to make these results. These results may indicate that text-mined GBLUP doesn’t seem to be effective in the case of traits that are more genetically affected by polygenic effect than causal variant effect. We also added this finding in the discussion (line 398). Thank you again for your comment.

Reviewer #2: In author's reply, authors mentioned a potential reason for the results that prediction using tm_SNP only gives lower accuracy is "text-mining didn’t identify all causal variant.". If this is the reason, GWAS using tm_SNPs may cause lower power because causal variants excluded from tm_SNPs are not tested. In terms of prediction, significant differences are rarely observed, and it looks like a significant difference is observed for CWT, but "text_mined + excepted" is worse than "evenly_extracted + excepted". I would recommend authors to run more cross validation to further verify their conclusion. A paired t test may also be considered.

Thank you for pointing this out. We totally agree that if text-mining can’t identify all causal variant, the GWAS using tm_SNPs may have lower power than using whole SNPs. However, the lower accuracy of tm_SNPs only seems to be more affected by the loss of polygenic effect (e.g gene x gene, gene x SNPs etc) than missing the causal variant. Even though the text-mining misses a small number of causal variants (variant recall will be decreased.), it still seems good way to filter out a large number of common SNPs (variant precision will be increased.!). According to this description, this method may have lower power to identifying the genetic architecture of target traits. However, considering the ability to remove the SNPs that make noise to GWAS with LD, it seems to be a good method for finding true causal variants of target traits.

In terms of the genomic prediction results, as you commented on, only CWT and D_SF showed significant differences (by t-test). The reason why em_SNPs showed higher accuracy in CWT is seem to be because CWT is more affected by polygenic effect than causal variant effect due to the characteristic of quantitative traits (or carcass trait). We added these results at table 6, and discussion for these results also added (line 398). Thank you again for the time and effort that you dedicated to providing feedback on our manuscript.

**Table 6. Accuracy of evenly-mined GBLUP and text-mined GBLUP**

| **Traits** | **exp_777k + tm_SNPs** | **exp_777k + em_SNPs^1^** |
| --- | --- | --- |
| **CWT** | 0.451 ± 0.01 | 0.471 ± 0.01 |
| **BF** | 0.421 ± 0.01 | 0.419 ± 0.01 |
| **EMA** | 0.437 ± 0.004 | 0.438 ± 0.01 |
| **S_SF** | 0.129 ± 0.03 | 0.099 ± 0.02 |
| **D_SF** | 0.189 ± 0.03 | 0.095 ± 0.02 |
| **S_IMF** | 0.168 ± 0.02 | 0.147 ± 0.02 |
| **D_IMF** | 0.225 ± 0.02 | 0.202 ± 0.03 |

**exp_777k + em_SNPs^1^**: multi-GRM GBLUP with evenly-mined SNPs and except SNPs.
