# Supplementary material for: Genome-wide identification of major genes and genomic prediction using high-density and text-mined gene-based SNP panels in Hanwoo (Korean cattle)
Source: PLoS One. 2020 Dec 2;15(12):e0241848. doi: 10.1371/journal.pone.0241848 (PMC7710051; doi:10.1371/journal.pone.0241848)
Supplement: Attachment — Submitted filename: Response_to_reviewer.docx [file pone.0241848.s005.docx]

Reviewer #1: The manuscript has been improved and my previous concerns were addressed. I have one more comment:
It is very useful but also very challenge to weight a set of genetic markers to improve prediction accuracy. A recent study developed a new method to improve the prediction accuracy by weighting the contributions of genetic markers in the prediction model (Lilin Yin et. al, Genome Biology, 2020). It should be nice if this could be discussed to compare with your strategy.

Response: Thank you for introducing a good method. We also agreed that weighting genetic markers is a challenge and further study is required. Therefore, we revised line 405 included at discussion to "Therefore, attempts to incorporate text mining into genomic predictions seem valuable and further study (i.e., other SNP effects weighting methods) using text mining can be expected to present the significant results.". In addition, the paper you recommended and (Wang H et al., Genetics Research, 2012) paper were referenced at the end of the sentence.

Reviewer #2: I have no further comments.

Response: Thank you for the time and effort you made during the whole review process.
